# Detection of Foodborne Pathogens by Surface Enhanced Raman Spectroscopy

**DOI:** 10.3389/fmicb.2018.01236

**Published:** 2018-06-12

**Authors:** Xihong Zhao, Mei Li, Zhenbo Xu

**Affiliations:** ^1^Research Center for Environmental Ecology and Engineering, Key Laboratory for Green Chemical Process of Ministry of Education, Key Laboratory for Hubei Novel Reactor and Green Chemical Technology, School of Environmental Ecology and Biological Engineering, Wuhan Institute of Technology, Wuhan, China; ^2^School of Food Science and Engineering, South China University of Technology, Guangzhou, China

**Keywords:** SERS, foodborne pathogens, rapid detection, nanoparticles, food safety

## Abstract

Food safety has become an important public health issue in both developed and developing countries. However, as the foodborne illnesses caused by the pollution of foodborne pathogens occurred frequently, which seriously endangered the safety and health of human beings. More importantly, the traditional techniques, such as PCR and enzyme-linked immunosorbent assay, are accurate and effective, but their pretreatments are complex and time-consuming. Therefore, how to detect foodborne pathogens quickly and sensitively has become the key to control food safety. Because of its sensitivity, rapidity, and non-destructive damage to the sample, the surface enhanced Raman scattering (SERS) is considered to be a powerful testing technology that is widely used to different fields. This review aims to give a systematic and comprehensive understanding of SERS for rapid detection of pathogen bacteria. First, the related concepts of SERS are stated, such as its work principal, active substrate, and biochemical origins of the detection of bacteria by SERS. Then the latest progress and applications in food safety, from detection and characterization of targets in label-free method to label method, is summarized. The advantages and limitations of different SERS substrates and methods are discussed. Finally, there are still several hurdles for the further development of SERS techniques into real-world applications. This review comes up with the perspectives on the future trends of the SERS technique in the field of foodborne pathogens detection and some problems to be solved urgently. Therefore, the purpose is mainly to understand the detection of foodborne pathogens and to make further emphasis on the importance of SERS techniques.

## Introduction

Foodborne illnesses caused by foodborne pathogens, such as *Salmonella, Vibrio parahaemolyticus, Listeria monocytogenes, Escherichia coli* O157: H7, and *Shigella*, led to a serious public health problem in food safety throughout the world. People are infected with foodborne illnesses that may cause a symptom of diarrhea or even death (Havelaar et al., 2015). In recent years, the incidence rate of microorganism is 61.92%, which mainly refers to *V. parahaemolyticus, Salmonella, E. coli* O157:H7, and so on. In 2015, the national food safety risk assessment center received 176 reports of foodborne outbreaks including food poisoning through the foodborne disease outbreak monitoring report system, affecting 2,065 people, including 11 deaths. Of these incidents, 97 were caused by food production, processing, and business operations, affecting 1,533 people, of whom 1 was killed; 79 of them were caused by non-food production and processing operations and 532 were sick, of whom 10 were killed (Yang et al., 2017). Additionally, according to the Centers for Disease Control and Prevention (CDC) 2016 estimates, foodborne diseases active surveillance network (FoodNet) identified 24,029 cases, 5,512 hospitalizations, and 98 deaths caused by confirmed or culture-independent diagnostic tests (CIDTs) positive-only infections. The largest number of confirmed or CIDT positive-only infections in 2016 was reported for *Campylobacter* (8,547), followed by *Salmonella* (8,172), *Shigella* (2,913), *E. coli* (1,845), *Cryptosporidium* (1,816), *Yersinia* (302), *Vibrio* (252), *Listeria* (127), and *Cyclospora* (55). The proportion of infections that were CIDT positive without culture confirmation in 2016 was largest for *Campylobacter* (32%) and *Yersinia* (32%), followed by *E. coli* (24%), *Shigella* (23%), *Vibrio* (13%), and *Salmonella* (8%). The overall increase in CIDT positive-only infections for these six pathogens in 2016 was 114% (range = 85–1,432%) compared with 2013–2015. Among infections with a positive CIDT result in 2016, a reflex culture was attempted on approximately 60% at either a clinical or state public health laboratory. The proportion of infections that were positive was highest for *Salmonella* (88%) and *E. coli* (87%), followed by *Shigella* (64%), *Yersinia* (59%), *Campylobacter* (52%), and *Vibrio* (46%) (Kapaya et al., 2018). Among them, *Salmonella* is the main cause of the global infection, and the risk of children is much higher than that of the adult (Ao et al., 2015). From the above analysis and summary, a rapid and effective method of the detection of foodborne pathogens should be urgently needed to guarantee the food safety and human health.

The traditional methods to detect foodborne pathogens are different according to the different requirements. The common and widespread method is to inoculate micro-organisms on the agar plate. First of all, we should cultivate microbes for several days, and then the microorganisms can be differentiated according to the corresponding physiological and biochemical features when they are cultured. From the above, it is these tedious processes that limit the extensive application of the method in reality. On the one hand, it will take a lot of time to identify bacterial pathogens and operate the complicated experiment. On the other hand, it is difficult to accurately tell some pathogenic bacteria with extremely similar physical and chemical features (Du et al., 2003). At present, with the rapid development of detection technology of foodborne pathogens, the technique is moving rapidly from traditional cell culture to modern detection technology, such as immunoassays technique (Chemburu et al., 2005), biosensor (Pedrero et al., 2009), PCR (Guan et al., 2013), gene probe (Shigemura et al., 2005), as well as impedance method (Cady et al., 1978), and so on. We have summarized their advantages and disadvantages in detail in **Table 1**. It can be seen from the table that although these techniques with good rapidity and effectiveness, these techniques are unable to detect bacterial pathogens at low concentrations (Zhao et al., 2014). Obviously, different detection methods have their own advantages and disadvantages, as reported by Cho and Ku (2017). Therefore, there is an urgent need for a detection method that has the advantages of minimum sample pretreatment, low cost, high sensitivity, good repetitiveness, and good on-site interpretation. The most important thing is that it can detect the lower concentration of the sample as easily as possible.

**Table 1:** Emerging technologies for the detection of pathogens.

Technique	Method	Advantages	Disadvantages
Immunological methods	Enzyme-linked immunosorbent assay (ELISA)	High specificity and sensitivity	Complex and narrow detection span, some cross-reaction
	Immunomagnetic separation assay (IMS)	Efficient and good specificity	Higher cost
	Immunoblot technique (IBT)	High resolution and high sensitivity	Complex operation
Metabolic technology	Microcalorimetry	Strong versatility and applicability	Long cycle and weak heat signal
	ATP bioluminescence technology	Fast, easy, and high sensitivity	It is difficult to distinguish between microbial and non-microbial ATP
	DNA probe	Specificity and fast, accurate	The added markers are hard to solve
	PCR, Multiplex, PCR	Reliable and efficient, linearity	Error due to non-target DNA amplification
Molecular biology technology	Quantitative PCR	Automated and real-time, high accuracy	Demanding equipment and expensive fluorescent probes, photo bleaching
	Isothermal amplification	High sensitivity and specificity	Difficult primer design
	microarray technique	High throughput and high efficiency, automated	Gene chip preparation and testing costs are high
	Electrochemical biosensors	Simple and good repeatability	Homogeneous sample necessary
Biosensor-based methods	Optical biosensors	Fast and real-time, label free	High equipment requirements
	Piezoelectric biosensors	Automated and high sensitivity	High equipment requirements

Spectroscopy techniques have been applied to the detection of foodborne pathogens in food, such as Raman spectrum, infrared spectroscopy, fluorescence spectroscopy, and other spectral detection techniques. One of them is the infrared spectroscopy that identifies the pathogenic bacteria from the food in order to obtain “fingerprints map.” And then the map will be compared and analyzed to get the composition of the bacterial cell wall and the information of target from the molecular characteristics of specific peaks. Ultimately, we can base on the above message to distinguish between different foodborne pathogens. So it is of great significance to study the mechanism of the infrared spectrum to establish a rapid detection method (Alvarezordóñez and Prieto, 2012). However, infrared spectrum is unsuitable to detect the samples in solution conditions, which causes it not to give full play to its detection advantages (Lu et al., 2011). Similarly, Raman spectroscopy also relies on the inelastic scattering of excitation light and molecular resonance to generate “fingerprints map,” which can be used to get the information of specific biomolecules. Raman spectroscopy is a real-time detection method that has the ability to quickly and effectively detect a variety of chemical structures and material composition (Craig et al., 2013). Compared with Raman spectroscopy, the detection range of infrared spectroscopy is narrower. Raman spectroscopy can provide more specific and readable biological or chemical information than the infrared spectrum over a wide range of laser wavelengths. But Raman spectroscopy has been less widely used than infrared spectroscopy due to its relative weak signals, fluorescence interference, and high cost of equipment. Hence, there is an urgent need for a better way to detect foodborne pathogens.

**FIGURE 1:**
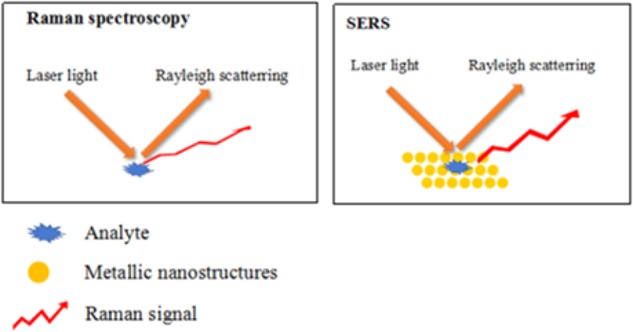
Schematic illustration of the difference between Raman spectroscopy and SERS.

As a result, the surface enhanced Raman spectroscopy is developed during more widespread and broader applications in different fields. This technology combining the advantages of infrared spectroscopy and Raman spectrum can not only detect samples in solution, but also it provides higher discrimination and sensitivity by using noble metal nanostructures to enhance the low-concentration single molecule Raman signal to several orders of magnitude (typically 10^7^ to 10^14^) (Sengupta et al., 2006). It is found that weak Raman scattering signals are greatly enhanced using noble-metal nanoparticles, while fluorescence is suppressed, as shown in **Figure 1**. Therefore, the identification and detection of targets by SERS has drawn much attention recently due to its high efficiency, sensitivity, and stability in aqueous solution. Because SERS can let some molecules to be adsorbed to the surface of some rough metal (such as gold and silver), which make them interact between each other to greatly enhance the signal of the Raman spectroscopy (Najafi et al., 2014). This special phenomenon is called surface enhanced Raman spectroscopy. It is because of its good and specific characteristics that the SERS was widely applied into the food industry (Zheng and He, 2014), pesticide residue (Liu et al., 2013), medical field (Han H.W. et al., 2009), environment (Woo et al., 2012), and so on. For example, Xie et al. (2017) combined the hybrid molecularly imprinted polymer with SERS to obtain a novel biosensor to detect chloramphenicol in milk. In addition, Jiang et al. (2018) used Ag-nanorod arrays for the detection of chemical residues in food oil via SERS. From the above-related research papers, SERS is still a hotpot today.

Nevertheless, although many targets combined with nanoparticles are enhanced by SERS, how to clearly specify of these signals of the observed vibrational signatures in the SERS and to determine the cellular mechanisms that explain the specific vibrational characteristics of these targets remains a problem. Zeiri et al. (2004) prepared silver colloid mixed with bacteria to explore the strong and highly specific Raman signal of various chemical components. The results showed that it was related to the biochemical components in bacteria, such as DNA, carboxylates, and perhaps phosphates. Premasiri and Ziegler (2010) equally credited the molecular origin of these signals to molecular components at the outer layer of the cell walls. In addition, some other inquiry experiments have been done, but the specific mechanisms for molecular enhancement have not yet been fully understood.

Consequently, if we want to understand how SERS is able to detect foodborne pathogens more quickly and effectively, we need to look through some relevant concepts in general. For instance, Stöckel et al. (2016) recently reported a review on how to quickly and reliably detect microorganisms under different conditions, including real-life environments and different parts of the body fluids in human. Liu et al. (2017) also summarized the latest trends and developments in the detection of pathogens based on labeled and label-free SERS. But there are few related literatures about the identification and detection of pathogenic bacteria by SERS in food due to the fact that the food components interfere with the signal of the bacteria. As a result, this paper comprehensively reviews the current trends and developments of pathogen detection by SERS and summarizes the achievements. This paper is mainly aimed at talking about the detection of bacteria, which contains related progress of working principle, signal source, and active substrate. The advantages and limitations of SERS in detecting foodborne pathogens are also discussed here. In addition, SERS combined with other technologies will be highlighted. Finally, we will mention some potential future developments of SERS technique in the field of foodborne pathogens and some problems that need to be solved urgently.

## The Background and Principle of Sers

Initially, vibrational spectroscopy methods, such as infrared spectroscopy (Randall, 1927) and Raman spectroscopy (Raman, 1928; Raman and Krishnan, 1928), were introduced in the early 20th century and have quickly developed as rapid and non-destructive tools in different fields. However, due to its weak inelastic scattered signals, so this technique compared to infrared spectroscopy was less applied. It was not until 1974 that Fleischmann and others discovered that pyridine molecules were absorbed onto the roughened metallic surface, causing a significant enhancement of the Raman signals by many orders of magnitude (Fleischmann et al., 1974). But they did not realize that was a new physical phenomenon at that time. Subsequently, Van Duyne and his team found through experimentation and systematic calculations that there are six orders of magnitude increasing relative to common Raman scattering signal of molecular in water environment, which was called SERS (Jeanmaire and Duyne, 1977). Not long after that, it is also due to the discovery of the SERS, which makes it quickly applied to various fields and has become a hot spot of research in all walks of life.

Since the late 1990s, SERS technology has been applied to the food detection, which mainly promoted by the need for fast and sensitive tools to detect food contaminants. In fact, the main application of SERS in food science at that time was to detect chemical and microbiological hazards rather than analyzing food components (Li and Church, 2014). SERS is currently used in food analysis which includes thiram (Guo et al., 2015), melamine (Lang et al., 2015), and ciprofloxacin (Xie et al., 2013a). Compared to traditional HPLC, GC and Raman, SERS is still an emerging technology in food analysis. Although the application of SERS is less relative to them, its application has increased a lot. Several years later, SERS has also been developed as a diagnostic tool for the detection of foodborne microorganisms through Raman fingerprinting, such as *Salmonella* (Chen et al., 2017), *Staphylococcus aureus* (Zhang et al., 2015), and *E. coli* (Pandey et al., 2017).

Despite the Raman signal of various components has been detected through SERS, some explanations of the observed vibration signatures of these signals have not yet been clarified. Thus, how to clearly specify the molecular origin of the vibrational signatures that appear in the SERS spectra of bacterial cells and to determine the cellular mechanisms that explain these species-specific vibrational signatures have been attracted much attention. Efrima and Zeiri (2010) discussed why SERS can be used to detect bacteria and the main reasons causing some differences between the spectra of several bacteria. SERS can be used to detect bacteria mainly due to its good Raman signal and can be used to detect single molecule level. The two main reasons that cause the differences of Raman signal are the internal or external differences of cells, which implied the production of the colloids of cellular interior or the component of cell wall (“external”). Premasiri and Ziegler (2010) discovered the SERS spectra of bacterium mainly related to adenine on the cell wall. Kubryk et al. (2016) combined a stable isotope approach with SERS to study the origin of the band at around 730 cm^-1^ in the SERS spectra of bacteria, which was assigned to adenine-related compounds. Premasiri et al. (2016) again studied the difference of Raman spectra of bacteria at 785 nm related to adenine, hypoxanthine, xanthine, guanine, uric acid, and AMP. Therefore, by judicious selecting the appropriate excitation wavelength and SERS-active media (e.g., gold and silver), and understanding the pre-resonance and non-resonance conditions of various cellular components, the repeatable Raman spectrum of bacteria can be simply and effectively obtained.

There have been several investigations related to the use of SERS for the detection of bacteria. The first report of bacterial SERS spectra by Efrima and Bronk (1998) used silver colloid substrate to detect *E. coli* and explain that the major bands are related to peptides and polysaccharides in cell walls and their membranes. Latest reports have shown that SERS can be used for bacterial identification under different conditions from those reported by Efrima. In addition, because *Bacillus* and *Clostridium* species is the presence of a large proportion of dipicolinic acid (DPA), in order to rapidly detect bacterial spores at low concentrations and to distinguish them better, the DPA can be used as a potential biomarker for the detection of spores. Zhang et al. (2005) used a low-cost SERS biosensor to detect the bacterial spores by targeting the DPA biomarker.

**FIGURE 2:**
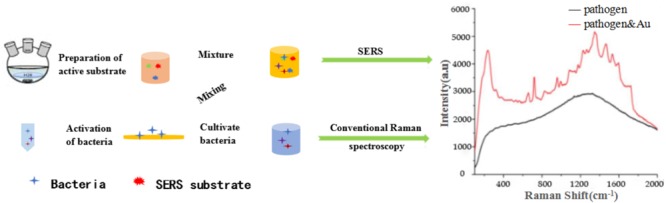
Schematic illustration of the overall procedure for SERS detection of pathogens combined with active substrates.

Thus, SERS mainly refers to as SERS substrates, which are necessary for SERS measurements and their SERS activity. SERS mainly refers to the use of the rough metal surfaces or metallic nanoparticles through special preparation, in a certain excitation region, the sample interacts with the special preparation of well-performing SERS substrates to enhance Raman signal of the surface or near molecular (Meheretu et al., 2014), as shown in **Figure 2**. However, the molecular mechanism of the SERS enhancement has not been clearly demonstrated. At present, there are two broad categories of SERS mechanisms commonly accepted: chemical enhancement mechanism (Gersten et al., 1979) and physical enhancement mechanism (Gersten and Nitzan, 1980). The Raman signal of SERS signal is closely related to the material of active substrate. The preparation of a good SERS active substrate is precondition for obtaining a more efficient Raman signal. Different active substrates have different enhancement effects on the samples. For example, the material of active substrate (Kleinman et al., 2013), the shape and size of the nanoparticles, the number of probes adsorbed on the active substrate, and the distance between them all affect the enhancement efficiency of SERS (Zhang et al., 2012).

## Active Substrate of Sers

Surface enhanced Raman spectroscopy is a novel technology of detection and analysis with high sensitivity, high testability, and good structural information of molecules through the characteristic peaks of Raman spectrum. Therefore, whether SERS is able to be developed an analysis method applied to practical application in the future, the key to the study of SERS is to prepare active substrates with high sensitivity, good stability, good reproducibility, and high selectivity.

At present, the most all-pervading and traditional active substrates are noble metal colloids (Rycenga et al., 2010), roughened noble metal surfaces (Moskovits, 1978), nanosphere self-assembly (Haynes and Duyne, 2016), template-directed deposition (Hyunhyub and Tsukruk, 2008), core-shell nanomaterial (Shen et al., 2013), and so on. Among them, the noble metal colloids in suspensions or aggregation are used mostly for SERS detection because of their low cost, easy preparation, and wide range of materials. However, on the one hand, the storage of noble metal colloids compared with nanostructured metal surfaces is complex, but also the nanoparticles were easy to gather and precipitates are unstable. The particle size of nanoparticles is poorly controlled. The method of template-directed deposition is easy to control the distance and improve the reproducible of nanoparticles, but it is not easy to save and its manufacture of roughness is troublesome. The signal to noise ratio of the film substrate is low. The spherical nanomaterial prepared by self-assembly is easily affected by the array and the diameter and spacing of nanomaterial (Huang et al., 2015; Li et al., 2017). The advantages of rough metal surfaces are that they are easy fast to operate, but they are easily absorbed by other substances, affecting results accuracy of SERS detection (Zheng et al., 2016). In a word, various SERS active substrates have their benefits and their shortcomings. We should be based on the requirement of a different environment to choose an optimal active substrate for SERS measurement. Here, we take colloid-based substrates as an example.

There are some advances in colloid-based substrates including the preparation of multi-component nanoparticles, such as Au-coated ZnO nanorods (Sinha et al., 2011), AgNP-coated amino-modified polystyrene microspheres (Zhao et al., 2013), Au-core shell silica nanoparticles (Au@SiO_2_ nanoparticles) (Quyen et al., 2013), silver-coated gold nanoparticles (Au@AgNPs) (Murshid et al., 2013), β-cyclodextrin coated SiO_2_@Au@Ag core/shell nanoparticles (Lu et al., 2015), porous Au–Ag alloy nanoparticles (Liu et al., 2016), and rhodamine derivatives (RhD) grafted Au@Ag core–shell nanocubes (CSNs) (Li et al., 2018). Nanoparticles with extremely high SERS activity and uniform surface morphology have been reported. For example, Au@SiO_2_ nanoparticles are synthesized with various silica shell thicknesses to form core/shell nanoparticles, then it was applied to detect Rhodamine 6G, which was found to show a good enhancement of SERS signal (Quyen et al., 2013). In the same way, in order to improve the stability of SERS active substrate in various environments, Au@AgNPs are synthesized to exhibit different Raman activities and stability by controlling the uniform deposition of gold and minimizing galvanic replacement. The Raman enhancement of Au@AgNPs is stronger than single nanoparticles (Murshid et al., 2013).

**FIGURE 3:**
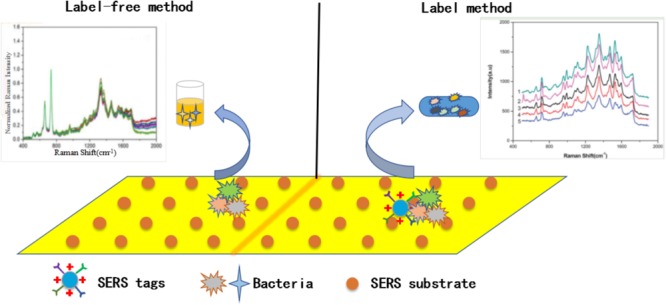
Schematic illustration of label-based and label free-based SERS method for bacteria detection.

On the one hand, different reporter molecules are selected to have different effects on the activity of SERS active substrates, such as organic dye. On the other hand, in order to obtain a stable and reproducible active substrate, different surface coatings need to choose, which will affect the activity of SERS active substrate (Wang et al., 2013c). Besides, with some special methods or materials, the activity of the active substrate can also be greatly improved. For example, with the high efficiency and accuracy of the microfluidic system, Li and Zhang prepared a low-cost and feasible SERS active substrate by depositing nanoparticles on a paper substrate and successfully detecting Rhodamine 6G at a low concentration (Li et al., 2013; Zhang et al., 2014). According to its characteristics, there are mainly two ways: label method and label-free method, as shown in **Figure 3**.

## Sers for Pathogen Bacteria Detection

### Label Method

**FIGURE 4:**
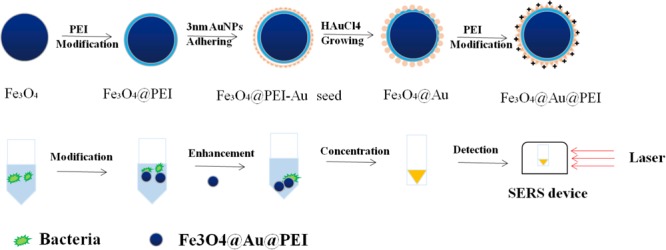
Schematic illustration of the detection process of SERS tags with Fe_3_O_4_@Au@PEI as active substrate.

In recent years, the differential detection of bacteria based on SERS requires many labeled elements, such as peptides, antibodies, and carbohydrates (Wang G. et al., 2010). This method that nanoparticles are modified by specific Raman reporting molecules and target recognition elements has a high Raman activity in sensitivity. This can also be called an indirect detection of target molecules by SERS. The preparation of SERS tags for bacterial detection requires multiple steps, including the design of SERS active substrates, the attachment of reporter, and the design of surface coatings and surface ligands, as shown in **Figure 4**.

Lin and Hamme (2014) modified gold nanoparticles with the help of Rhodamine 6G to obtain specific active SERS substrates. It was the first time that this method was applied to detect multiple drug-resistant *Salmonella* DT104 by SERS, and then the active substrate was characterized through electron microscopy to acquire the TEM image of *Salmonella*. The results showed that it was better to compare photo thermal response of the hybrid nanomaterial than single-walled carbon nanotubes (SWCNTs) and gold nanoparticles. Through this research approach, it can increase its great potential for rapid detection and photo-thermal therapy of clinical samples. Zhang H. et al. (2016) fabricated ordered hierarchical micro/nanostructured arrays with monolayer colloidal crystals as masks. They controlled the experimental conditions to synthesize different the morphology of hierarchical micro/nanostructured arrays, and then 4-aminothiophenol was added into active SERS substrate to measure the signal of surface Raman spectrum. By comparing different morphologies of nanostructured arrays and optimizing them, nanostructured arrays with better Raman signals were obtained. This provides a new direction for the preparation of active substrates and gets a higher Raman signal.

Penn et al. (2013) developed a kind of antibody-modified membrane which was used to act a function of SERS immunoassay of nanoparticle. The thin layer of gold was deposited on a polycarbonate track etched film, the capture antibody was immobilized on the surface of the gold plating film by coupling chemistry to serve as a capture substrate. And the target and Raman reporter were transported to the capture substrate via a syringe. The membrane was used to identify the feasibility of SERS-based immunoassay. In this article, an SERS-based immunoassay assay conducted on a membrane filter that implements flow was the first time to be applied to enhance Raman signal and significantly shorten the time of experiment.

In order to better capture the target and maintain the stability of the nanoparticles, Wang J. et al. (2016) used label-free method for the detection of bacterial pathogens by SERS. Based on the combination of polyethylenimine-modified Au-coated magnetic microspheres (Fe_3_O_4_@Au@PEI) and concentrated Au@Ag nanoparticles, which were called the capture-enrichment-enhancement (CEE) three-step method. Subsequently, this active substrate based on SERS was used to detect microorganisms in tap water and milk samples as well as to measure the minimum limits of *E. coli* and *S. aureus*. On the one hand, the results show that this method is not only suitable for general detection, but the Raman signal of detection by SERS is higher than before. On the other hand, the detection of foodborne pathogens by three-step method shows that the method has the advantages of shorter measuring time, simple operation steps, and higher sensitivity than the previously reported method based on SERS for the detection of bacteria. Wang J. et al. (2016) synthesized an Au-coated magnetic nanoparticles core/shell nanocomposites with nanoscale rough surfaces, which was characterized by its highly uniform in size and shape. When used for the detection of *S. aureus*, this active substrate exhibits excellent and good SERS activity.

However, in order to further improve the detection sensitivity, there are many studies using a “sandwich-type” assay combined with SERS technology to detect pathogens. At first, the antibody is immobilized on the surface of the substrate (i.e., the immunological substrate). And then the bacteria to be detected in the solution were specifically captured by the immunological substrate. Finally, the nano-metal sols labeled with the antibody and the Raman probe carried out immune recognition (Dong et al., 2014; Hou et al., 2014). The Raman signal of the testing molecules is obtained through the SERS.

Wang et al. (2011) have done a lot of research on immunoassay and obtained progress. In 2011, they used silica-coated magnetic nanoparticles as a magnetic probe to form a sandwich assay, which was applied into the detection of foodborne pathogens by SERS, such as *Salmonella* and *S. aureus*. What is more, the limit of peanut and spinach which were included in multiple pathogen detection were detected by SERS. Wang et al. (2013b) designed a specially reduced the antibody half-fragment. The signal of SERS was greatly improved by the interactions of sandwiched antibody–antigen to provide an efficient and convenient means for SERS immunoassay platform and expand the application for SERS. Based on SERS, Han X.X. et al. (2009) established a specific method using a sandwich-based immunoassay to set up the gold–protein–gold (Au/Au) and gold–protein–silver (Au/Ag) sandwiches assay, which resulted in high reproducibility of SERS signal. It was also found that the latter signal of SERS was stronger than the former about seven times, due to the different contributions of the two metal layers to the SERS. This kind of sandwiched structure provided higher sensitivity and repeatability for the detection by SERS.

On the one hand, though the label-based SERS has the advantage of higher sensitivity and repeatability and can be applied to the detection of multiplex pathogenic bacteria, organic dye may bring about the message of microbes lost. On the other hand, the addition of organic dyes may increase the experimental analysis time. In addition, the preparation of SERS tags is complex and costly. Therefore, there is an urgent need to develop rapid, simple, and low-cost SERS methods.

### Label-Free Method

The label-free SERS method is a direct detection method. Because there is no special need to add into active substrate during the detection of targets by SRES, such as dye molecules. So Raman’s signal will not be disturbed by other components and the information obtained is more accurate and reliable. In addition, this kind of detection method is widely used because of its simple, rapid, low cost, and rich information of molecules detected by SERS. Here we primarily discuss two types of SERS substrates including their advantages and limitations of applications in detecting pathogenic bacteria, because they are the most common, the simplest active substrates.

#### Metallic Colloid-Based SERS Method

**FIGURE 5:**
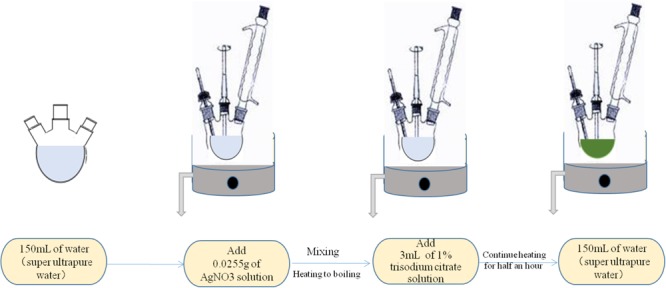
Schematic illustration of the method of preparation silver colloidal nanoparticles by chemical reduction.

The metallic colloid (such as gold and silver) is most widely used and studied for the SERS measurements. The common and effective synthesis method used for this type of nanoparticles is the chemical reduction, as shown in **Figure 5**. The method mainly depends on the interaction between the bacterial liquid and the SERS active substrate in solution to directly obtain the Raman signal of molecules. On the one hand, they are inexpensive and easily prepared. On the other hand, this method operated on the material from the molecular level, so that relatively uniform nanoparticles can be obtained. In addition, the reaction in the solution can better control the size and shape of nanoparticles. The rough preparation process is shown in **Figure 3**. Noble metal nanoparticles mainly including gold (Au) and silver (Ag) colloids in diameters between 10 and 200 nm are widely used to detect variable analytes.

Based on SERS, Cowcher et al. (2013) mixed silver nanoparticle colloids with the bacterial liquid for rapid detection of *Bacillus* and other pathogens. SERS is used to detect *Bacillus* in food because of its fast analysis speed and high sensitivity. Compared with the microscopy, the results showed that the SERS can more rapidly and efficiently detect DPA biomarker, which served as a marker of bacillus *in vivo*. The method has the advantage of being convenient, readable, and cheap. However, the limitation of this method is the extraction of the DPA from the spores. Mungroo et al. (2016) developed a microfluidic platform for the detection of pathogenic bacteria by SERS based on silver nanoparticle active substrates. SERS was combined with stoichiometry, principal component analysis, and linear discriminant analysis to effectively and quickly identify eight common species of foodborne pathogens. And then each species of pathogen was created a series of peak assignments which established a unique identification of each species, but it was still a bit difficult to identify Gram-negative and Gram-positive bacteria. However, this study provides a good reference for the detection of multiple pathogens in the food industry. Xie et al. (2013b) mixed different batch gold colloid with seven different bacteria to distinguish the foodborne pathogens. From the results, the gold colloid is beneficial to rapidly and sensitively detect foodborne pathogens through the SERS.

According to the above description, most of studies focus on the SERS effect form single metal nanoparticles, so there are some shortcomings; for example, the poor enhancement effect of single gold nanoparticles and the poor stability and homogeneity of silver. In order to better integrate their advantages, there are also metal nanocomposite for the measurement of target by SERS. For instance, many nanolayer shells on Ag cores (such as Ag/carbon, Ag/Au, and Ag/SiO2) were fabricated to improve the Ag nanostructure’s time-stability. Khlebtsov et al. (2015) prepared uniform Au@Ag core/shell cuboids and dumbbells with controllable Ag shells of 1–25 nm in thickness, which provided a comparation of new active substrates for targets in low concentrations with high SERS response of internal molecules in core/shell metal nanostructure. Stated thus, metallic colloids are widely used for the detection of foodborne pathogens by changing its preparation conditions, shape, and size. Although this particle is low in cost and easy to prepare, the stability of this particle is not good, which may have a certain impact on the results of the detection.

#### Nanostructured Metal Surface-Based SERS Method

As is well known to us, the metal nanoparticles with a good and stable Raman signal are cheap and easy to prepare. However, due to its poor repeatability, the application and development of metal nanoparticles is limited. Therefore, in order to improve the repeatability of active substrate for pathogenic bacteria detection, many researchers turned to the pathogens detection by SERS based on the nanostructured metal surface.

Wang Y. et al. (2010) assembled silver nanoclusters (AgNCs) to form spheres, which was used as SERS-active substrate with the diameter of 60–80 nm. As a result, it was found that not only the Raman signal of SERS can be enhanced to 10^8^, but also the sensitivity of the SERS detection was improved and outstanding repeatability was achieved. What is more, three kinds of pathogens, as well as live and dead bacteria, were also detected and classified by SERS based on the special active substrate. With in-depth study of the active substrate, it provides the possibility the detection of single cell. Kee et al. (2013) used a plasmonic nanohole sensor to quickly, efficiently, and quantitatively monitor the bacterial growth and antibiotic sensitivity. The plasmonic nanohole arrays were fabricated by a mask-based deep ultraviolet lithography method and were measured the characteristic of bulk refractive index sensitivity and surface mass sensitivity. With *E. coli* as an experimental object, the effect of the plasmonic nanohole sensor was tested. According to analysis, *E. coli* was specifically captured and rapidly grew on its surface. In addition, the sensor was found to be able to detect antibiotic sensitivity rapidly and efficiently within 2 h. These studies are beneficial to the clinical application of bacterial infection.

In 2009, by self-assembly technique for preparing solid substrate of SERS, Yan et al. (2009) developed in identification of three types of foodborne pathogens, which included *E. coli, Bacillus cereus*, and *S. aureus*. From the result, there was a strong reproducible SERS signal, and the signal not only came from small molecules, but also from the bacterial cells. A few years later, the researchers also studied the reasons for the SERS enhancement signals by using the self-assembly technique guided based on the template (Reinhard and Dal Negro, 2011).

Based on the SERS, Wu et al. (2013) prepared silver nanorod array substrates that were applied to detect the pathogenic bacteria in food, such as mung bean sprouts, spinacia oleracea, and romaine lettuce. By optimizing the length of silver nanorod array, the minimum value of Raman signal of pathogenic bacteria detected by SERS was obtained. At the same time, the mathematical analysis method was used to reduce the experimental operation cycle. These provide a powerful platform for SERS to detect foodborne pathogens at low concentrations and expand the prospect of practical applications. It also plays a solid foundation for the popularization and application of SERS technology.

Overall, variable active substrates have the weaknesses and strengths of different functions. Just like we need to choose different methods to detect bacteria, we should reasonably select the suitable active substrates for our test object to avoid unnecessary errors (Law et al., 2015).

## Other

The SERS is not only used for the detection of foodborne pathogens in foods, but also it has many applications in other aspects. Besides, there are also many improved methods to enhance the Raman signal of SERS active substrates, so as to realize the effective and rapid detection of different components by SERS.

In order to extend the application of SERS tags, recently several SERS-related multimodal probes had been developed. Park et al. (2009) combined functionalized gold nanorods with the biomarker molecules of the analyte to allow this SERS substrate to target and image the targets. For instance, SERS tags were integrated with fluorescence, which was a more novel study. Lin et al. (2014) used a novel graphene oxide to encapsulate gold nanoparticles and then functionalized it to obtain a good Raman signal. This active substrate was used to rapidly detect foodborne pathogens and sensitive Raman imaging of *S. aureus* and *E. coli*. In the same year, the institute had developed a fluorescent SERS dual-mode tag that combines the lanthanide-based upconversion nanoparticles with near-infrared lasers to achieve the first biological imaging of living cells and *in vivo*, which provides infinite possibility for medical applications in future (Niu et al., 2014; Zhang et al., 2014).

As for other applications, Wang et al. (2013a) based on 4-mercaptopyridine (4-MPY) to obtain functionalized silver nanoparticles, which were used for the detection of heparin through SERS. The limit of heparin was acquired in terms of sensitivity, selectivity, and linearity. The method exhibited satisfying results, which indicated a great practicality for application in real analysis and monitor other related fields. Based on lateral flow assay biosensor, Fu et al. (2016) developed a novel low-concentration quantitative analysis of a specific biomarker with SERS. The specific biomarker of selected human immunodeficiency virus type 1 DNA was sensitively and quantitatively measured successfully by labeled gold nanoparticles. Similarly, Wang J. et al. (2016) and Wang X. et al. (2017) used labeled active substrate to simultaneously detect dual DNA markers, which were related to Kaposi’s sarcoma-associated herpesvirus and bacillary angiomatosis. The result showed that Raman signal of target became more sensitive than previous studies. In addition, for medical research, it is very important for us to pay more attention to accurate analysis of specific biomarkers in clinical aspects. Cheng et al. (2017) combined magnetic beads with SERS tags to achieve the detection of the early diagnosis and treatment of cancer. The result demonstrated that SERS is promising for application in the accurate diagnosis of cancer.

In a word, SERS may become a powerful tool in any field in the future. What is more, this technology will continue to be developed and improved to apply to more aspects.

## Sers Combined With Other Technologies

At present, SERS has been applied in various fields; however, the molecular mechanism of the interaction between the active substrate and the sample to be tested is still unclear. In the past, the molecular enhancement mechanisms of SERS have been studied only by normal detection by SERS and theoretical modeling. Nowadays, Zhang M. et al. (2016) got a good result by combining SERS with isotope tracing to demonstrate the mechanism of surface chemical reactions. On the other hand, in order to further apply SERS, Xie et al. (2013b) used SERS to detect seven kinds of pathogenic bacteria to acquire the characteristic peaks of these pathogens, and combined with principal component analysis and cluster analysis to classify these pathogens to better distinguish the categories of pathogens.

It is well known that enzyme-linked immunosorbent assay (ELISA) is also a very universal method widely used to detect foodborne pathogens, because the main characteristic of ELISA is its high sensitivity. But one important and key aspect of this approach is that the method has a preprocessing process. If the pretreatment is not properly done, due to the complex diversity of the sample, the samples containing salt, acid, metal ions, and so on will affect the results of the detection by ELISA. SERS has also been widely used in biological systems with good selectivity and sensitivity. Therefore, Ko et al. (2015) combined the ELISA with the SERS to achieve direct detection of these samples without preprocessing and improve the sensitivity of detection. What is more, Wang W. et al. (2016) obtained the SERS activity of EC-SERS by combining the ultra-microelectrode with SERS in order to obtain real-time transient Raman information, and the combination has a more stable and high signal of SERS. Similarly, in order to detect the amount of residues in samples, Wang L. et al. (2017) combined molecular imprinting with SRES detection technology to finally obtain a good specificity and sensitivity of Raman spectroscopy signals, besides the time of detection by SERS is greatly shortened. Wang W. et al. (2016) studied the materials of SERS active substrate by using the electrochemical method to improve the stability and sensitivity of SERS.

In addition, there are various techniques associated with the SERS, such as high performance liquid chromatography (Hassanain et al., 2016), infrared spectroscopy (Chen et al., 2015), and thin-layer chromatography (Lv et al., 2015). It is because of this combination techniques, making the application of SERS greatly expanded and widely used in various fields. In addition, due to the poor reproducibility of the active substrate of the SERS technology, which limits its application to the detection of foodborne pathogens in foods for on site, while the microfluidic system called “Lap on a chip” has the advantages of low dosage, high efficiency, accuracy, and so on. The SERS-microfluidic system can overcome this shortcoming (Jung et al., 2007; Pu et al., 2017; Wang C. et al., 2017). The above shows that the purpose of the combined technology is to learn from each other and to achieve the optimization of the method, thus expanding the prospect of the application of the detection of SERS technology in various fields.

## Outlook

The current research has shown that SERS technology has been widely applied to the detection and identification of foodborne pathogens in food, but there are still several aspects to be further improved here. First of all, how should we pretreat the food sample before we are going to detect foodborne pathogens by SERS in food. Because the failure of the preprocessing process will affect the effect of the detection; secondly, for different types of active SERS substrate, there are many influencing factors, such as the size and shape of active substrate. How should we better control these factors to improve Raman signal? In addition, how can we better combine the SERS with other technologies? Finally, there is no researcher to collect and summarize the fingerprint and analysis information of pathogenic bacteria of SERS, so as to create a shared network platform and database for search and operation. If the “Raman fingerprinting” database and network sharing platform for many kinds of microorganisms are established, it will be able to cross the boundaries of time and region to realize the sharing of resources among multiple research institutions and improve the efficiency of scientific research. The researchers will be able to directly obtain the fingerprint of the known microorganism and match it with the microbial information identified by the traditional method, which can achieve fast and effective detection of foodborne pathogenic bacteria by SERS and solve a series of related food safety issues caused by foodborne pathogens. Therefore, how to solve these problems quickly and effectively is one of the future directions for SERS development.

## Conclusion

After two decades of development, SERS has been widely used in various fields. With the progress of the times, this trend of development will not be weakened. For the detection of foodborne pathogens based on SERS technology is universally paid attention to. Therefore, the application of this technology to the detection of foodborne pathogens will still be a hot spot. What is more, SERS technology can achieve stable, reliable, rapid, and sensitive identification of pathogenic bacteria in food. People can set up fast, simple, specific, sensitive, and low-consumption detection by SERS according to the theory and application practice of SERS technology. Although the detection technology by SERS has not been totally found their way into products, but with the improvement of micro-fabrication technology and nanotechnology, Raman spectroscopy has been also continuously miniaturized. The emergence of a variety of portable and hand-held Raman spectrometers has made it possible to detect foodborne pathogens directly and rapidly on site. Therefore, the prospects of SERS development are unlimited.

## Author Contributions

XZ and ML wrote the manuscript. XZ, ML, and ZX revised the manuscript critically for important intellectual content. All authors read and approved the final manuscript.

## Conflict of Interest Statement

The authors declare that the research was conducted in the absence of any commercial or financial relationships that could be construed as a potential conflict of interest.
